# A comparison of muscle activation and concomitant intermuscular coupling of antagonist muscles among bench presses with different instability degrees in untrained men

**DOI:** 10.3389/fphys.2022.940719

**Published:** 2022-09-06

**Authors:** Lejun Wang, Minjie Qiao, Haifeng Tao, Xiaoqian Song, Qineng Shao, Ce Wang, Hua Yang, Wenxin Niu, Yiqing Chen

**Affiliations:** ^1^ Sport and Health Research Center, Physical Education Department, Tongji University, Shanghai, China; ^2^ Engineering Research Center of Clinical Translational Digital Medicine, School of Biomedical Engineering, Shanghai Jiao Tong University, Shanghai, China; ^3^ School of Medicine, Tongji University, Shanghai, China

**Keywords:** unstable training, bench press, muscle activation, intermuscular coupling, phase synchronization index

## Abstract

The aim of this study was to analyze and compare the muscle activation and concomitant intermuscular coupling of antagonist muscles among bench presses with different instability degrees. Twenty-nine untrained male college students performed bench press exercises at an intensity of 60% 1 RM on three conditions: small unstable bench press with Smith machine (SBP), medium unstable bench press of free weight (FWBP), and large unstable bench press with increased instability by suspending the load with elastic bands (IIBP). One-way repeated measures analysis of variance was used to compare integrated EMG activity values of the biceps brachii (BB), posterior deltoid (PD), long head of the triceps brachii (TB), anterior deltoid (AD), upper portion of the pectoralis major (PM) muscles, and phase synchronization index (PSI) of BB-TB and PD-AD antagonist muscle pairs. A higher integrated EMG of BB muscle was found during bench press with a more unstable condition. IIBP showed a higher integrated EMG of prime movers (TB, AD, and PM) and stabilizing of BB than SBP and FWBP. PSI between muscle pairs of BB-TB in the gamma frequency band was higher in SBP than the other bench presses with unstable conditions, which may be related to the optimal “internal model” for antagonist muscles during bench press exercise. Therefore, IIBP training may be an effective accessory exercise to maintain a higher level of muscle activation across primary and stabilizing muscles with a lighter load for untrained men, while SBP may be a suitable bench press exercise for untrained participants who have not developed the neuromuscular adaptations necessary for correct stabilization of the elbow joint.

## 1 Introduction

In recent decades, it has become more and more popular to perform health-oriented training with instability challenges (Behm and Anderson, 2006; Behm et al., 2010). It has been assumed that resistance training with instability results in higher muscle activities and thus is more beneficial to health and strength performance (Schick et al., 2010; Williams et al., 2020; Zemková et al., 2021). However, previous research has provided mixed results, and resistance training performed under unstable conditions may produce greater, no change, or even decreased muscle activation compared to traditional training (Behm et al., 2002; Anderson and Behm, 2004; Saeterbakken et al., 2011; Saeterbakken and Fimland, 2013; Dunnick et al., 2015; Lawrence et al., 2021). The varying results may be related to the type of instability device used, the difference in workload, the function of the muscle, and other relevant factors (Nairn et al., 2015; Gołaś et al., 2017; Saeterbakken et al., 2017; Dugdale et al., 2019; Garcia-Lopez et al., 2020). Specifically, when performing instability resistance training, superior strength training gains have been mainly found in trained populations, while a few studies have revealed no significant improvement in strength and power between stable and unstable resistance training for untrained populations (Mate-Munoz et al., 2014; Ghigiarelli et al., 2018). Considering that comparison studies of muscle activation during stable and unstable resistance training have been mainly conducted on participants with training experience (Gołaś et al., 2017; Garcia-Lopez et al., 2020), we wonder whether instability conditions may change muscle activation during training for untrained subjects.

Bench press is a classic exercise that is commonly accompanied by measuring or training upper-body strength (Schick et al., 2010). When performing bench press exercises, several methods have been adopted to create instability. First, compared to a Smith machine bench press lifting the bar in a fixed path, the free weight bench press may offer instability in all three planes of motion, which force the lifter to contract the muscles in a more natural fashion to keep balance while exerting force at an inconsistent velocity (Cotterman et al., 2005; Schick et al., 2010). Moreover, instability could be further increased by using a specially designed flexible barbell (e.g., Bandbell bar, “Earthquake” bar, or multi-grip Swiss Bar), suspending the load from a barbell through the use of elastic bands as well as completing the bench press on an unstable surface (Saeterbakken and Fimland, 2013; Dunnick et al., 2015; Ostrowski et al., 2017; Costello, 2020). In previous studies, the differences in muscle activation (as reflected by EMG amplitude) between a Smith machine and free weight bench presses and between free weight and increased instability bench press have been compared and revealed (Cotterman et al., 2005; Schick et al., 2010; Dunnick et al., 2015; Saeterbakken et al., 2016). As the instability degree from a Smith machine to free weight and to increased instability bench press increases gradually, muscle activation during the three bench presses may differ due to the change in instability degree. However, few studies have compared muscle activations of the three types of bench press concurrently.

Generally, unstable resistance exercises are performed to increase the activity of stabilizing muscles (mainly antagonistic muscles for bench press exercise) to enhance joint stiffness and overcome the instability induced by unstable loads (Anderson and Behm, 2004; Kohler et al., 2010; Ostrowski et al., 2017). From the viewpoint of motor control, the coordination control of agonist and antagonist muscles plays a vital role in human movement adjustment (van Dieën et al., 2003; Oomen et al., 2015). As previous researchers mainly analyzed muscle activations of stabilizing muscles and primary movers of bench press exercise separately and mainly for trained participants (Schick et al., 2010; Ostrowski et al., 2017), current literature struggles to provide a concrete conclusion on the muscle activation and concomitant intermuscular coupling of antagonist muscles among bench presses with different instability degrees, especially for untrained populations.

EMG–EMG synchronization analysis has been widely used to explore the intermuscular coupling of co-contracted muscles (Farina et al., 2014; Wang et al., 2019). In previous studies, the synchronization of co-contracted muscle oscillations has been mainly evaluated in the frequency (coherence analysis) and phase (phase synchronization analysis) domains (van Asseldonk et al., 2014; Wang et al., 2015; Pizzamiglio et al., 2017). In particular, EEG and EMG signals have been revealed to be phase locked (Mima et al., 2000; Ushiyama et al., 2011), which demonstrates that phase synchronization activities of EMG signals between co-contracted muscles may reflect cortical-related modulation information (Wang et al., 2015). Moreover, it has been mathematically revealed that coherence and phase coupling are closely related as phase coupling is both necessary and sufficient to yield non-zero coherence while the coherence is determined by both the amplitude and phase coupling (Bruns, 2004).

The aim of this study was to analyze the effects of different degrees of instability on muscle activation and concomitant intermuscular coupling of antagonist muscles in bench press exercise in untrained men. Bench presses with three different instability degrees were adopted: small unstable bench press with Smith machine (SBP), medium unstable bench press of free weight (FWBP), and large unstable bench press with increased instability by suspending the load with elastic bands (IIBP). It was hypothesized that muscle activation and intermuscular coupling of antagonist muscles would be influenced by instability conditions of bench press and may follow a monotonic increasing trend from SBP to FWBP to IIBP.

## 2 Materials and methods

### 2.1 Subjects

Twenty-nine healthy untrained male college students (age 19.4 ± 2.1 y, height 174.2 ± 4.3 cm, weight 63.0 ± 7.0 kg, and bench press 1 RM 44.6 ± 5.5 kg) volunteered to participate in this study. None of the subjects had any systematic training experience in upper-body training experience, and they were refrained from vigorous exercises 24 h before the experiment. All subjects were fully informed of the experimental procedures and potential risks before signing voluntary written consent. The experiment was approved by the Ethics Committee of Tongji University.

### 2.2 Data recording

#### 2.2.1 Experimental setup

A familiarization session was conducted for the first visit to the laboratory before the start of the test to introduce the experimental protocol and provide an opportunity for the subjects to use the three types of bench presses adopted in this study. Each subject was first asked to conduct a dynamic warm-up consisting of 2-min jogging, 4-min upper-body stretching, and 2 sets of 5 repetitions of 40% perceived 1 RM workload bench press on the Smith machine. During the familiarization session, subjects performed a series of bench press exercise tests at 40%, 60%, and 80% perceived 1 RM workload for 2 sets of 3 repetitions with three types of bench presses used in this research study. A 5-min rest was provided between each set. The formal experiment was conducted after more than 2 days.

The formal experiment comprised two sessions. Each subject first conducted the same dynamic warm-up as described earlier. During the first testing session (second visit), subjects performed a 1 RM bench press with a standard barbell and typical load according to the National Strength and Conditioning Association’s guidelines for maximal strength testing (Association and Miller, 2012). A self-selected load bench press warm-up was first performed, the workload of which allowed each subject to finish 6–10 repetitions (approximately 50% predicted 1 RM) of the exercise. Then, after 1–5 min of rest, subjects then select a weight based on the previous effort which allows them to perform three repetitions (approximately 80% predicted 1 RM)**.** Subjects then progressively increased resistance by 5–10% of the previous attempt. The 1 RM was determined within five attempts, and a 3–5-min rest period was provided between each attempt. Only one 1 RM test was conducted to prescribe load for all three different bench presses due to each bench press using the same condition (SBP) and percentage (60%) of 1 RM. The second test session was arranged at least 7 days after the 1 RM bench press test.

During the second session (third visit), subjects conducted the tests of three types of bench press exercises. Three types of bench presses were performed in the order of SBP, FWBP, and IIBP and were separated by at least 1 h between the two consecutive tests. Each type of bench press was performed for one set of five repetitions. Before each type of bench press test, a 10-min dynamic warm-up was performed as described earlier. There was no food or beverage, but water was offered, and the same electrodes were used throughout the whole testing session.

**FIGURE 1:**
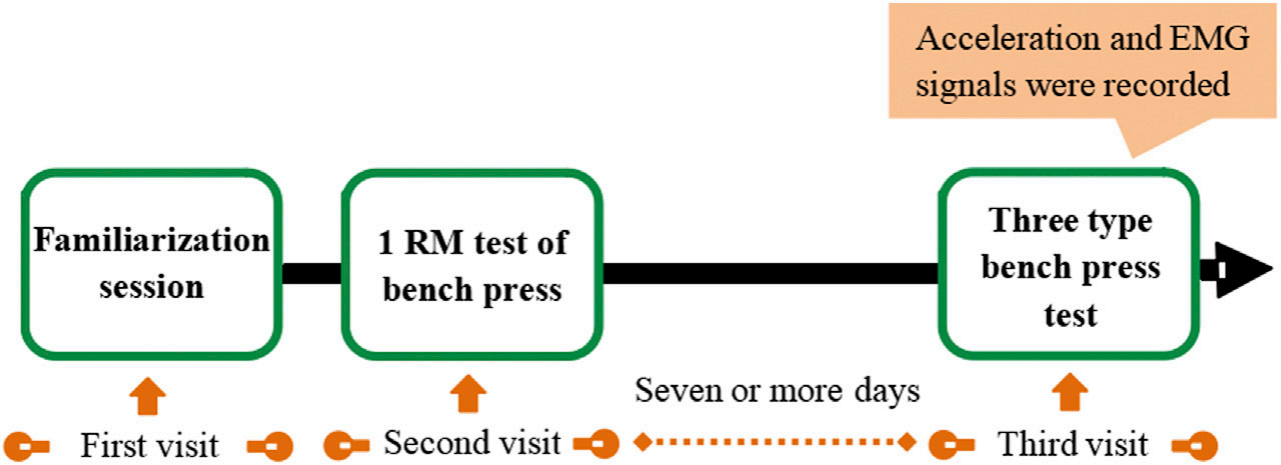
Experimental protocol.

During the three different bench press exercises, triaxial acceleration data of barbell and surface EMG signals of the right biceps brachii (BB), posterior deltoid (PD), long head of the triceps brachii (TB), anterior deltoid (AD), and upper portion of the pectoralis major (PM) muscles were recorded synchronously. The experimental protocol was depicted in Figure 1.

#### 2.2.2 Bench press test

**FIGURE 2:**
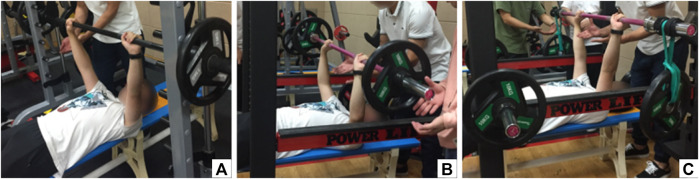
Example diagram of performing bench press for three different conditions. **(A)** Small unstable bench press with Smith machine (SBP), **(B)** medium unstable bench press of free weight (FWBP), and **(C)** large unstable bench press with increased instability (IIBP).

Figure 2 showed an example diagram of performing bench press for three different bench press conditions. SBP was carried out on a Smith machine (Matrix Fitness, Johnson Health Tech, Cottage Grove, MN, United States). Subjects were instructed to perform the bench press following a fixed “straight up and down” route. Subjects were required to lie supine on a bench with their legs slightly apart and upper bodies parallel to the ground. The position of the bench was individually adjusted so that the shank was perpendicular to the thigh, and the whole soles of the feet were in contact with the ground. Subjects grasped the barbell at a comfortable width, which met the requirement of placing it slightly wider than the shoulder width. The hand spacing of each subject was recorded for replication in subsequent FWBP and IIBP tests. The elbows performed flexion comfortably within the coronal, and the wrists were kept in a neutral position. Upon verbal command, subjects concentrically pushed the barbell until executing full elbow extension and then immediately eccentrically lowered the barbell until the chest was touched, approximately 3 cm superior to the xiphoid process (Orange et al., 2019). To maximize external validity, lifting cadence was determined by the tempo that each subject felt was most natural to him (Schick et al., 2010), with a 2-s rest between the two successive repetitions. The attempt was not considered valid once the subjects’ head, upper back, and buttocks left the bench or both feet off the ground. The barbell was not permitted to bounce off the chest.

FWBP was executed in a standard Smith squat rack (Pro-Elite Strength Systems, Salt Lake City, UT, United States). All subjects conducted FWBP without any assistive devices or additional unstable disturbances. The testing procedure was identical to the SBP. Both the load intensity (60% 1 RM) and the testing posture of the FWBP were the same as SBP.

For the IIBP test to create extra instability on the basis of free weight bench press, an unstable load (as provided by a flexible barbell and a load suspended by elastic bands) was applied. Elastic bands were linked with specific weights (60% 1 RM-15 kg) of barbell plates and attached to both ends of the barbell. Subjects completed the IIBP using the same techniques and requirements as FWBP.

#### 2.2.3 Acceleration measurement

Acceleration (ACC) signals were measured from three orthogonal axes (X, Y, and Z corresponding to mediolateral, anteroposterior, and vertical directions, respectively) by using a triaxial accelerometric sensor (Kinv TS) fixed to the right edge of the barbell. To ensure accurate results, the X-, Y- and Z-axes were consistently perpendicular to the sagittal, coronal, and horizontal planes, respectively. The calibration of acceleration was conducted along all three axes prior to the beginning of each individual measurement. All triaxial acceleration data were continuously sampled at 100 Hz. Acceleration signal recordings were performed synchronously with the EMG measurements.

#### 2.2.4 Electromyography measurement

The electromyography (EMG) signals were recorded from the right biceps brachii (BB), posterior deltoid (PD), long head of the triceps brachii (TB), anterior deltoid (AD), and upper portion of the pectoralis major (PM) muscles using a wireless EMG system (BTS FREEEMG 1000, BTS, Garbagnate Milanese, MI, Italy) with a 2.0 cm inter-electrode distance. To obtain proper electric contact and low inter-electrode resistance, the skin was prepared at the electrode locations by shaving, lightly abrading with fine emery paper, and cleaning with alcohol wipes. Electrodes were positioned along the longitudinal axis of each muscle according to SENIAM (http://seniam.org/). Specifically, the electrodes were placed as follows: on the BB at the line between the medial acromion and the fossa cubit at 1/3 from the fossa cubit, on the PD at the area about two fingerbreadths behind the angle of the acromion, on the TB long head at 50% on the line between the posterior crista of the acromion and the olecranon at two finger widths medial to the line, on the AD at 1.5 cm distal and anterior to the acromion, and on the PM upper portion at the midclavicular line over the second intercostal space (Rodriguez-Ridao et al., 2020). Signals were converted from analog to digital with a sampling rate of 1,000 Hz.

### 2.3 Data processing and analysis

#### 2.3.1 Integrated acceleration amplitude calculation

The acceleration signals during rest were discarded, and signals for each bench press repetition were acquired. Acceleration signals recorded from X-, Y-, and Z-axes were then band-pass filtered at 0.8–50 Hz offline by means of a 4^th^-order zero-phase-shift Butterworth filter and were full-wave rectified. Following full-wave rectification, the acceleration signals of each axis were root mean squared with a 50-ms moving rectangular window to create a linear envelope. The integrated acceleration amplitude of each axis was calculated for each repetition and was averaged over the five repetitions.

#### 2.3.2 Integrated electromyography activation calculation

The EMG signals recorded from BB, PD, TB, AD, and PM were band-pass filtered at 5–500 Hz using a 4^th^-order zero-phase-shift Butterworth filter and were full-wave rectified. Based on the method of previous research (Aagaard et al., 2002), muscle activation was defined, and EMG signals of muscle activation were acquired for each bench press repetition. The integrated EMG (iEMG) activation was calculated referring to the following formula and was averaged over each repetition:

$$\mathrm{iEMG}=\overset{n}{\underset{i=m}{\Sigma }}\left|\mathrm{EMGDat}a_{i}\right|\times \;\Delta \mathrm{t,}$$ (1)

where m is the start point of each bench press repetition, n is the endpoint of the corresponding repetition, $\mathrm{EMGDat}a_{i}$ is the EMG amplitude of the ith sample point, Δt is the interval time between two conservative sampling points, and i represents the order number of the dealing sample point.

#### 2.3.3 Electromyography–electromyography phase synchronization analysis

EMG signals were selected over the period in which the bench press was performed. The selected EMG data were filtered for the frequency range 8–12 Hz (alpha band), 15–35 Hz (beta band), and 35–60 Hz (gamma band) by means of a 4^th^-order zero-phase-shift Butterworth filter. Phase synchronization index (PSI) in alpha, beta, and gamma frequency bands between EMG of synergistic (AD-TB and AD-PM) and antagonist (BB-TB and AD-PD) muscle pairs were calculated as follows:

$$\mathrm{PSI}=\sqrt{{\langle \cos\theta_{\mathrm{xy}}^{H}\left(t\right)\rangle }_{t}^{2}+{\langle \sin\theta_{\mathrm{xy}}^{H}\left(t\right)\rangle }_{t}^{2}},$$ (2)

where $\cdot_{\text{t}}$ means the average of all the values.

$$\theta_{\mathrm{xy}}^{H}\left(t\right)\;=n\theta_{x}^{H}\left(t\right)\;-m\theta_{y}^{H}\left(t\right),$$ (3)

where $\theta_{x}^{H}\left(t\right)$ is the phase angle calculated based on the Hilbert transform of EMG signal recorded from one muscle and $\theta_{y}^{H}\left(t\right)$ from another muscle (one-to-one correspondence following the muscle pairs above). The values of m and n were all assigned to 1 according to previous research studies (Quiroga et al., 2002).

Data processing was performed using MATLAB R2019a software (Mathworks, United States).

### 2.4 Statistical analysis

Statistical analysis was performed using SPSS 13.0 for Windows (SPSS). Normality was tested by means of the Kolmogorov–Smirnov test. Integrated acceleration amplitude, integrated EMG activation, and PSI in alpha, beta, and gamma frequency bands for the SBP, FWBP, and IIBP groups were all tested to follow a normal distribution (*p* > 0.05). Therefore, a repeated measures analysis of variance [within factors: bench press type] was used to determine the significance of acceleration amplitude, integrated EMG activation, and PSI in three frequency bands among the SBP, FWBP, and IIBP groups. A posthoc test of Bonferroni was used to determine differences among pairs of means. Effect sizes were evaluated using partial eta-squared, which can be calculated by $SS_{\mathrm{effect}}/\left(SS_{\mathrm{effect}}\;+\;SS_{\mathrm{error}}\right)$ , where $SS_{\mathrm{effect}}$ is the sum of squares of an effect for one variable and $SS_{\mathrm{effect}}$ is the sum of squares error in the ANOVA model. The effect size was considered small if partial eta-squared < 0.06, medium if partial eta-squared < 0.14, and large if partial eta-squared > 0.14 (Cohen, 2013). All significant thresholds were fixed at α = 0.05.

## 3 Results

### 3.1 Integrated acceleration amplitude

**FIGURE 3:**
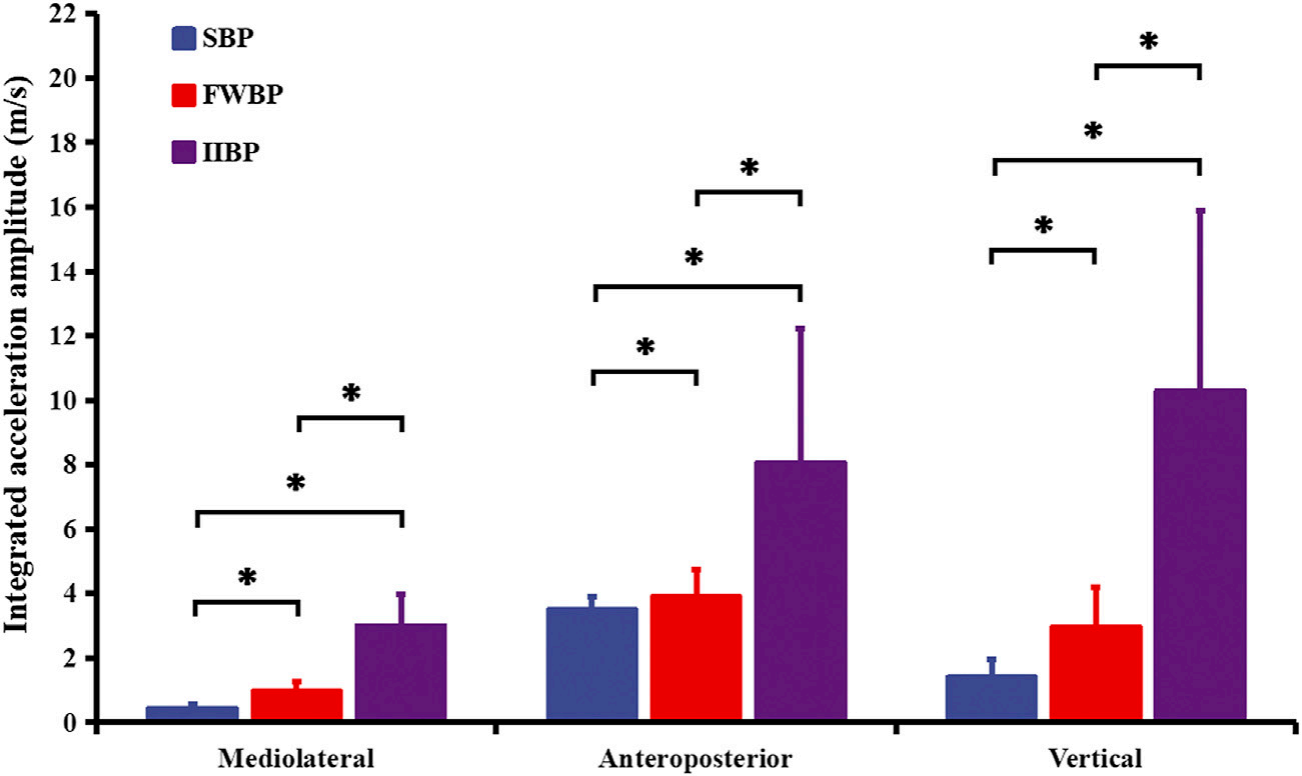
Integrated acceleration amplitude in mediolateral, anteroposterior, and vertical directions for three different bench press conditions. Results were expressed as mean ± SD. Significant differences are indicated by asterisks (*p* < 0.05).

Figure 3 displays the integrated acceleration amplitude in mediolateral, anteroposterior, and vertical directions for three different bench press conditions. Significant main effects of bench press type (SBP, FWBP, and IIBP) on integrated acceleration amplitude were found in mediolateral, anteroposterior, and vertical directions (mediolateral: F = 441.480, *p* = 0.000, partial eta-squared = 0.940; anteroposterior: F = 439.804, *p* = 0.000, partial eta-squared = 0.940; vertical: F = 219.346, *p* = 0.000, partial eta-squared = 0.887). A higher integrated acceleration amplitude was found during bench press with a more unstable condition at mediolateral (SBP-FWBP: *p* = 0.000; SBP-IIBP: *p* = 0.000; FWBP-IIBP: *p* = 0.000), anteroposterior (SBP-FWBP: *p* = 0.000; SBP-IIBP: *p* = 0.000; FWBP-IIBP: *p* = 0.000), and vertical (SBP-FWBP: *p* = 0.000; SBP-IIBP: *p* = 0.000; FWBP-IIBP: *p* = 0.000) directions.

### 3.2 Muscle integrated electromyography activation

**FIGURE 4:**
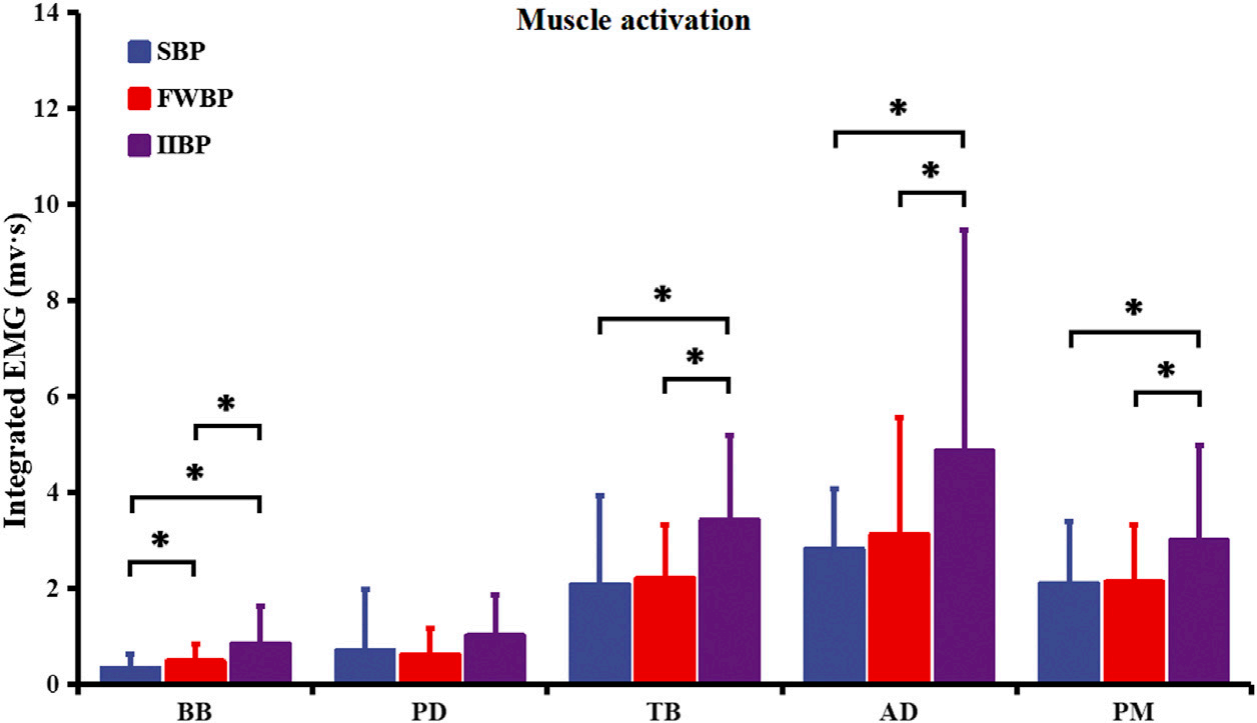
Integrated EMG activation of stabilizing muscles (BB and PD) and prime movers (TB, AD, and PM) for three different bench press conditions. Results were expressed as mean ± SD. Significant differences are indicated by asterisks (*p* < 0.05).

Figure 4 represents integrated EMG activation of stabilizing muscles (BB and PD) and prime movers (TB, AD, and PM) for three different bench press conditions. Significant main effects of bench press type (SBP, FWBP, and IIBP) on integrated EMG activation were found in the stabilizing muscle of BB and prime movers of TB, AD, and PM (BB: F = 21.701, *p* = 0.000, partial eta-squared = 0.598; TB: F = 38.817; *p* = 0.000, partial eta-squared = 0.822; AD: F = 11.159; *p* = 0.001, partial eta-squared = 0.625; PM: F = 17.894; *p* = 0.000, partial eta-squared = 0.744). For stabilizing muscle of BB, a higher integrated EMG was found during bench press with a more unstable condition (SBP-FWBP: *p* = 0.002; SBP-IIBP: *p* = 0.000; FWBP-IIBP: *p* = 0.001). For prime movers of TB, AD, and PM, IIBP showed a higher integrated EMG of prime mover than SBP (TB: *p* = 0.000; AD: *p* = 0.008; PM: *p* = 0.001) and FWBP (TB: *p* = 0.000; AD: *p* = 0.003; PM: *p* = 0.000), with no significant integrated EMG activation between the FWBP and SBP groups (TB: *p* = 0.763; AD: *p* = 0.580; PM: *p* = 1.000).

### 3.3 Electromyography–electromyography phase synchronization index

**FIGURE 5:**
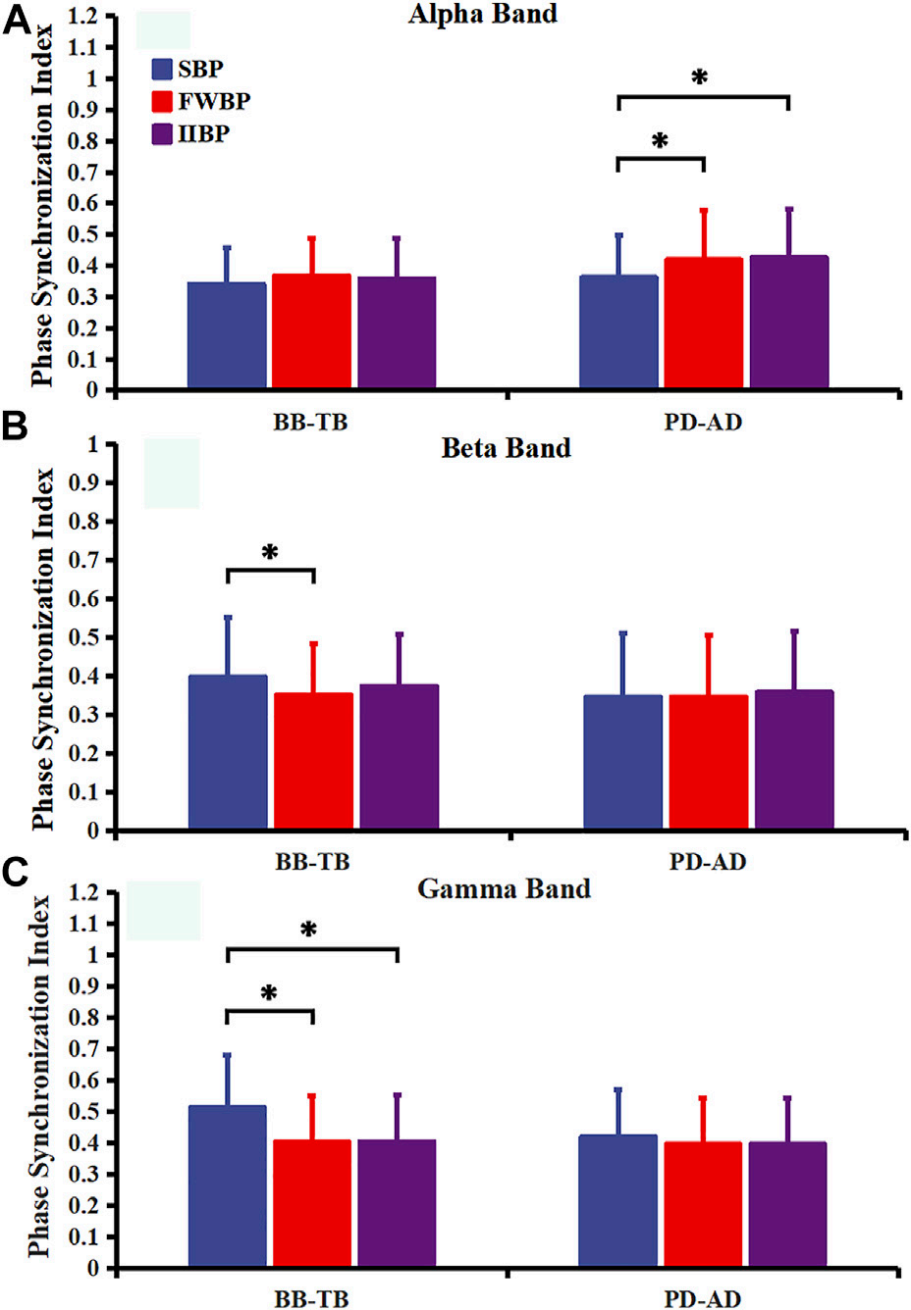
PSI in alpha **(A)**, beta **(B)**, and gamma **(C)** frequency bands between EMGs of antagonist muscle pairs (BB-TB and PD-AD) for three different bench press conditions. Results are expressed as mean ± SD. Significant differences are indicated by asterisks (*p* < 0.05).

Figure 5 represents PSI in three frequency bands between EMGs of antagonist muscle pairs BB-TB and PD-AD for three different bench press conditions. Significant main effects of bench press type (SBP, FWBP, and IIBP) on PSI value were found in PD-AD muscle pair in the alpha frequency band (F = 6.201, *p* = 0.004, and partial eta-squared = 0.900). PSI of PD-AD in the alpha frequency band was significantly higher in FWBP (*p* = 0.035) and IIBP (*p* = 0.006) groups than in the SBP group. For beta frequency band, significant main effects of bench press type on PSI value were found in the BB-TB muscle pair (F = 4.275, *p* = 0.019, and partial eta-squared = 0.893). PSI of BB-TB in the SBP group was significantly higher than the FWBP group in the beta frequency band (*p* = 0.031). In gamma frequency band, significant main effects of bench press type on PSI value were found in the BB-TB muscle pair (F = 24.009, *p* = 0.000, and partial eta-squared = 0.907). PSI of BB-TB (SBP-FWBP: *p* = 0.000; SBP-IIBP: *p* = 0.000) showed a significantly higher value in SBP than the other bench presses with unstable conditions.

## 4 Discussion

A comparison of the effects of bench presses with different instability degrees found that, overall, the instability condition [small unstable (SBP), medium unstable (FWBP), and large unstable (IIBP)] played a significant role in both muscle activation and intermuscular coupling of antagonist muscles during bench press exercise. It seemed that the changes in the results according to the increase in instability may vary depending on the roles of the muscles in the movement (i.e., prime mover and stabilizing muscles). For the BB muscle (stabilizing muscle), a higher integrated EMG was found during bench press with a more unstable condition. However, muscle activation of prime mover and intermuscular coupling did not follow a monotonic changing trend with the increase in instability as was originally predicted. Generally, IIBP with the large unstable condition showed a higher integrated EMG of the prime mover than the SBP and FWBP groups, with no significant integrated EMG activation between SBP and FWBP. PSI between antagonist muscle pair BB-TB in gamma frequency band showed a significantly higher value in SBP than the other bench presses with the medium and large unstable conditions. As far as we know, this is the first study to examine differences in muscle activation and intermuscular coupling of antagonist muscles among bench presses with different instability degrees.

Previous researchers have suggested that resistance training under unstable conditions was designed to preferentially increase the activity of stabilizing muscles (antagonist muscle) to enhance joint stiffness and overcome the instability induced by unstable loads (Anderson and Behm, 2004; Williams et al., 2020). In the current research, acceleration amplitudes in mediolateral, anteroposterior, and vertical directions, as well as integrated EMG activation of BB (stabilizing muscle), concurrently showed an increasing linear trend from SBP to FWBP to IIBP as predicted. In previous studies, bench press under unstable conditions has been found to mainly increase activation of the BB muscle compared to stable bench press (Ostrowski et al., 2017; Costello, 2020). The previous results may lend evidence to support the viewpoint of previous research studies and partially verify our research hypothesis (Lawrence et al., 2017; Lawrence et al., 2021). On the other hand, activation of stabilizing muscle PD showed no significant difference between stable bench press (SBP) and unstable bench press (FWBP and IIBP) in this study, which is consistent with previous research studies (Ostrowski et al., 2017; Lawrence et al., 2021). In fact, AD, MD, and PM may also play a role as stabilizing muscles in maintaining shoulder stability, although the effects may be varied according to bench press intensity (Costello, 2020). It has been suggested that PD is a small stabilizing muscle accompanying AD and MD and opts not to be influenced by instability conditions during bench press exercise (Lawrence et al., 2017; Ostrowski et al., 2017).

Referring to the influence of instability on muscle activation of prime movers during bench press, however, previous research struggled to provide consistent conclusions, especially for untrained populations (Saeterbakken et al., 2011; Daniels and Cook, 2017). In previous studies, muscle activation of prime movers between free weight and Smith bench press and between free weight and instability bench press has been compared for trained participants (Schick et al., 2010; Saeterbakken et al., 2011; Dunnick et al., 2015). It seemed that the activation of prime movers opted not to be influenced by unstable conditions. For example, Schick et al. (2010) revealed that there were no differences in muscle activation for AD and PM between the Smith machine and free weight bench press at both lower (70% 1 RM) and higher (90% 1 RM) intensities. In the research of Dunnick et al. (2015), upper-body muscle activation is not different in the bench press between stable and unstable conditions at two different (60% and 80% 1 RM) intensities. In this study, integrated EMG activation of prime movers (TB, AD, and PM) showed no significant difference between the SBP and FWBP groups, which is consistent with previous research studies (Schick et al., 2010; Dunnick et al., 2015; Costello, 2020). However, integrated EMG activation of prime movers (TB, AD, and PM) for the IIBP group was all significantly higher than the SBP and FWBP groups, which differed from those previously reported findings of no differences between stable and unstable loads.

It has been suggested that muscle activation during bench press exercises can be influenced by many factors such as instability degree, workload, etc (Nairn et al., 2015; Gołaś et al., 2017; Saeterbakken et al., 2017; Garcia-Lopez et al., 2020). In the current research, absolute workload (60% 1 RM tested with a standard barbell) was kept constant for the three types of bench press exercise, which may indicate a higher relative workload for bench press exercises with more instability (Saeterbakken et al., 2021). However, constant relative workloads determined by the respective 1 RM of each bench press have been mainly adopted in previous studies (Lawrence et al., 2017; Costello, 2020). A higher workload of bench press would increase muscle strength and power output as well as induce a higher muscle activation of prime movers (Schick et al., 2010). In addition, relevant previous research studies were mainly conducted on trained populations, while untrained men were tested in the present study (Saeterbakken et al., 2011; Saeterbakken and Fimland, 2013). As untrained subjects have no skilled bench press technology, especially for a higher unstable condition, which would influence muscle control and muscle activation in the current research. It can be convinced that the abovementioned differences in methodology provided more challenges for the neuromuscular system and allowed a generation of greater prime movers’ activation.

In terms of intermuscular coupling, PSI of antagonist muscle pair BB-TB in the gamma frequency band was significantly higher in the SBP group than that in the FWBP and IIBP groups. PSI in the gamma frequency band has been suggested to be closely related to the common neural inputs of the co-contracted muscles in strong isometric and dynamic voluntary contractions (Gwin and Ferris, 2012). The higher PSI found in the SBP group may indicate an increased descending common drive of the antagonist muscle pair BB-TB when performing SBP than bench press with unstable conditions. As prime movers and stabilizing muscles mainly performed dynamic contractions during bench press exercises, PSI in the gamma frequency band may reflect predominant central adjustment in this study. On the other hand, significant PSI of antagonist muscle pair PD-AD in the alpha frequency band was significantly lower in the SBP group than that in the FWBP and IIBP groups. Intermuscular oscillatory coupling in the alpha frequency band can be influenced by multifactors such as stretch-reflex, mechanical resonance, and cortical drives (McAuley and Marsden, 2000; Wang et al., 2015). It has been reported that the postural tremor of limb muscles may generate an oscillatory frequency of around 10 Hz (Saxton et al., 1995). Therefore, the higher PSI found in the FWBP and IIBP groups in the alpha frequency band may be closely related to the limb tremor induced by unstable conditions of bench press exercises.

It has been suggested that during movement practice and training, the central nervous system may learn to form a fully formed “internal model” of dynamics, in which the coactivation of antagonist muscles is controlled in a more economical coordination strategy (Osu et al., 2002; Gribble et al., 2003; Kattla and Lowery, 2010). Therefore, the decrease in antagonist coactivation and an increase in intermuscular coupling of antagonist muscles may indicate a more optimal motor control style. In this study, increased PSI in the gamma frequency band and decreased antagonist muscle coactivation level were found together in the stable bench press (SBP) when compared to bench presses under unstable conditions (FWBP and IIBP). In the current research, subjects were all untrained men without very skilled technology, especially for bench press with medium and large instability, which may bring challenges for movements and thus influence the coupling of antagonist muscles. It seems that there is a positive correlation between PSI value in the gamma frequency band and the existence of an optimal “internal model” for co-contraction muscles during bench press exercise. Therefore, the results may also indicate that unstable conditions during bench press would weaken the optimality of motor control style and decrease the coupling of antagonist muscles of elbow joint for untrained men.

However, limitations should also be acknowledged in the current research. First, bench press tests were conducted in the order of SBP, FWBP, and IIBP rather than in randomized order, which may have had a potential impact on the results. Second, it may be suggested that workload should be determined according to the 1 RM of the three different bench press exercises separately. It is indeed that the constant absolute workload adopted for the three types of bench press exercise may indicate a higher relative intensity for bench press with more instability, and this could be a reason to influence muscle activation and thus may obscure the influence of the researched factors (i.e., instability) on the observed indices (Saeterbakken et al., 2021). Third, in order to maximize external validity, subjects performed bench press exercises at their natural pace, which may induce different speeds of movement among the three types of bench press and influence muscle activation in the current study. Fourth, as the participants were young male untrained subjects, the insufficient familiarization session may have had a potential influence on the stability and reliability of the results. However, we have calculated the ICC of the muscle activation based on the EMG signals of the five repetitions of each type of bench press exercise. The ICC of SBP, FWBP, and IIBP averaged over the five tested muscles was 0.980 ± 0.007, 0.965 ± 0.045, and 0.984 ± 0.006. Moreover, there was a significant positive correlation between muscle activation of SBP-FWBP (r = 0.847 ± 0.045, with all of the *p* ≤ 0.001), SBP-IIBP (r = 0.775 ± 0.110, with all of the *p* ≤ 0.001), and FWBP-IIBP (r = 0.916 ± 0.040, with all of the *p* ≤ 0.001). Therefore, the results may indicate the reliability of the results in the current research. Fifth, data were only collected and analyzed from the upper limbs of the right side, and the influence of imbalance performance between the left and right sides has not been considered. Moreover, in this study, we only collected five main muscles according to previous research studies, and other muscles, such as the middle deltoid, upper trapezius, and latissimus dorsi, have not been tested and analyzed (Dunnick et al., 2015). Lastly, there is always an inherent risk of crosstalk when examining EMG activity. In this study, the electrodes were placed by the same experienced researcher according to the previous recommendations, and repeated measurements were conducted for each participant, which may be helpful in reducing the effects of crosstalk.

The results of the present study suggest that IIBP may lead to an increased requirement for stabilization of the elbow joint from muscles such as BB and for prime movers of both the elbow and glenohumeral joint from muscles of TB, AD, and PM. Therefore, IIBP training may be an effective accessory exercise to maintain a higher level of muscle activation across primary and stabilizing muscles with a lighter load for untrained men. Moreover, the results of the present study may also suggest that SBP may be a suitable bench press exercise for untrained participants who have not developed the neuromuscular adaptations necessary for correct stabilization of the elbow joint. However, future research should examine the differences in training-related changes in muscle strength and power development as a result of SBP, FWBP, and IIBP resistance training.

In conclusion, a higher integrated EMG of the BB muscle was found during bench press with a more unstable condition. Large unstable bench press with increased instability showed a higher integrated EMG of prime movers (TB, AD, and PM) than small unstable and medium unstable bench presses, with no significant integrated EMG activation between small unstable and medium unstable bench presses. PSI between antagonist muscle pair of BB-TB in the gamma frequency band showed a significantly higher value in small unstable bench press than in bench presses with medium and large unstable conditions, which may be related to the optimal “internal model” for co-contraction muscle during bench press exercise. Therefore, IIBP training may be an effective accessory exercise to maintain a higher level of muscle activation across primary and stabilizing muscles with a lighter load for untrained men, while SBP may be a suitable bench press exercise for untrained participants who have not developed the neuromuscular adaptations necessary for correct stabilization of the elbow joint.

## Data Availability

The raw data supporting the conclusion of this article will be made available by the authors, without undue reservation.

## Abbreviations

SBP, small unstable bench press with Smith machine; FWBP, medium unstable bench press of free weight; IIBP, large unstable bench press with increased instability; EMG, electromyography; iEMG, integrated electromyography; PSI, phase synchronization index; BB, biceps brachii; PD, posterior deltoid; TB, triceps brachii long head; AD, anterior deltoid; PM, pectoralis major upper portion; BB-TB, biceps brachii-triceps brachii; PD-AD, posterior deltoid-anterior deltoid; RM, repetition maximum; SD, standard deviation.
